# Further validation of the internet addiction test: Psychometric characteristics in a portuguese university sample

**DOI:** 10.1192/j.eurpsy.2021.1526

**Published:** 2021-08-13

**Authors:** C. Bento, A.T. Pereira, C. Marques, D. Mota, A. Macedo

**Affiliations:** 1 Institute Of Psychological Medicine, Faculty Of Medicine, University of Coimbra, coimbra, Portugal; 2 Cri Psiquiatria, Centro Hospitalar Universitário de Coimbra, Coimbra, Portugal; 3 Institute Of Psychological Medicine, Faculty Of Medicine, University of Coimbra, Coimbra, Portugal

**Keywords:** Internet addiction, psychometric properties, Portuguese adolescents

## Abstract

**Introduction:**

The Internet Addiction Test (IAT) is a 20-item, self-reported questionnaire that measures the presence and severity of Internet addiction which is an increasing problem in adolescents. Although a Portuguese version IAT has been validated in adults, its psychometric properties have never been evaluated before, in adolescents.

**Objectives:**

To analyse the reliability and construct and concurrent validity of the IAT in a Portuguese adolescent sample.

**Methods:**

772 adolescents (53.5% girls), mean aged 13.21±2.246, answered the Portuguese versions of the IAT and the Portuguese versions of validated scales to evaluate: Cyberbullying, Game Addiction, Agressivity and Anxiety, Depression Scales. To study the temporal stability, 377 (60.5% girls) respondents answered the questionnaires again after approximately four-six weeks. The total sample was aleatory splitted to realize the exploratory and the confirmatory factor analyses.

**Results:**

Exploratory and confirmatory factor analyses supported a second order two-factor structure - “Isolation and Social Commitment” and F2-“Negligence and Functional Commitment”. The χ2/df value was 2.260 and had a significant p value; it had the lowest RMSEA score = .074 (p< .001) and it had the highest TLI (.980) and CFI (.905). IAT mean scores were no different between genders [Girls=29.25±18.775 vs. Boys: 30.85±17.929, p=.405]. The Cronbach’s alphas were > .85. Pearson correlation between the test and the re-test was r=.660. The IAT, video game addiction (r=.434), Cyberbullying (r=.383), anxiety (r=.209) and depression (r=.263) were significantly correlated (p<.001).

**Conclusions:**

The Portuguese IAT has good reliability and validity, showing to be an adequate instrument for measuring Internet Addiction symptoms in Portuguese Adolescents.

